# Hyperoxia in Sepsis and Septic Shock: A Comprehensive Review of Clinical Evidence and Therapeutic Implications

**DOI:** 10.7759/cureus.68597

**Published:** 2024-09-04

**Authors:** Sharayu Paunikar, Vivek Chakole

**Affiliations:** 1 Anesthesiology, Jawaharlal Nehru Medical College, Datta Meghe Institute of Higher Education and Research, Wardha, IND

**Keywords:** critical care medicine, oxidative stress, oxygen therapy, septic shock, sepsis, hyperoxia

## Abstract

Sepsis and septic shock are leading causes of mortality in intensive care units, characterized by a dysregulated immune response to infection, leading to severe organ dysfunction. Oxygen therapy is a cornerstone of supportive care in sepsis management, aimed at correcting hypoxemia and improving tissue oxygenation. However, the administration of supplemental oxygen must be carefully managed to avoid hyperoxia, which can lead to oxidative stress and additional tissue damage. This review aims to comprehensively analyze the clinical evidence regarding hyperoxia in the context of sepsis and septic shock, evaluating its potential therapeutic benefits and risks and discussing the implications for clinical practice. A thorough literature review included observational studies, randomized controlled trials (RCTs), meta-analyses, and clinical guidelines. The review focuses on the pathophysiology of sepsis, the mechanisms of hyperoxia-induced injury, and the clinical outcomes associated with different oxygenation strategies. The evidence suggests that while oxygen is crucial in managing sepsis, the risk of hyperoxia-related complications is significant. Hyperoxia has been associated with increased mortality and adverse outcomes in septic patients due to mechanisms such as oxidative stress, impaired microcirculation, and potential worsening of organ dysfunction. RCTs and meta-analyses indicate that conservative oxygen therapy may be beneficial in reducing these risks, though optimal oxygenation targets remain under investigation. This review highlights the importance of careful oxygen management in sepsis and septic shock, emphasizing the need for individualized oxygen therapy to avoid the dangers of hyperoxia. Further research is required to refine oxygenation strategies, establish clear clinical guidelines, and optimize outcomes for sepsis and septic shock patients. Balancing adequate oxygenation with the prevention of hyperoxia-induced injury is crucial in improving the prognosis of these critically ill patients.

## Introduction and background

Sepsis and septic shock are severe medical conditions characterized by a dysregulated host response to infection, leading to life-threatening organ dysfunction [1]. According to the Sepsis-3 definition, sepsis involves an inflammatory response to infection that results in significant tissue damage and can progress to septic shock, associated with profound circulatory, cellular, and metabolic abnormalities [2]. Septic shock is typically marked by persistent hypotension requiring vasopressors to maintain adequate mean arterial pressure and a high serum lactate level, indicating cellular hypoperfusion despite adequate fluid resuscitation. These conditions are major contributors to mortality and morbidity in intensive care units (ICUs) worldwide, making their management a critical focus in critical care medicine [2]. Oxygen therapy plays a crucial role in the management of critically ill patients, including those with sepsis and septic shock. Oxygen therapy aims to ensure adequate oxygen delivery to tissues, thereby preventing cellular hypoxia and its associated complications. Maintaining optimal oxygenation is vital in sepsis, where patients often experience significant impairments in oxygen transport and utilization due to microcirculatory dysfunction and mitochondrial abnormalities [2]. Clinicians commonly use supplemental oxygen as an initial therapeutic intervention in sepsis management to enhance tissue oxygenation, especially during acute respiratory distress or shock episodes. However, the optimal oxygenation level remains controversial, particularly regarding the potential risks associated with both hypoxia and hyperoxia [3].

Hyperoxia, an excess of oxygen in the tissues, can occur when supplemental oxygen is administered in higher than necessary concentrations. While ensuring sufficient oxygenation is critical, hyperoxia can lead to adverse physiological effects, including oxidative stress, which results in the formation of reactive oxygen species (ROS) [3]. These reactive molecules can cause cellular and tissue damage, contribute to inflammation, and impair microcirculatory function. Hyperoxia can also induce pulmonary toxicity, decrease cardiac output by causing vasoconstriction, and impair cerebral blood flow, potentially exacerbating the clinical course of sepsis and septic shock [4]. The objective of this review is to comprehensively explore the clinical evidence regarding hyperoxia in the management of sepsis and septic shock, examining its potential therapeutic benefits and risks. By critically evaluating the current literature, this review aims to provide insights into the optimal use of oxygen therapy in sepsis, offering guidance on balancing the benefits of adequate oxygenation against the potential harms of hyperoxia. Additionally, it seeks to identify gaps in knowledge and suggest directions for future research in this critical area of critical care medicine.

## Review

Pathophysiology of sepsis and septic shock

Sepsis is a life-threatening condition defined by a dysregulated host response to infection, which leads to organ dysfunction. The sepsis pathophysiology involves a complex interplay between the immune system and inflammatory processes. Initially, the body's immune system responds to the infection by releasing pro-inflammatory cytokines and activating immune cells to eradicate the pathogens [5]. However, if the infection persists or becomes overwhelming, this response can become maladaptive. This maladaptive response often results in widespread tissue damage and can lead to multiple organ dysfunction syndrome (MODS). During sepsis, the immune system may shift from a hyper-inflammatory state to one of immune paralysis, increasing susceptibility to secondary infections. This dual state of hyperinflammation followed by immunosuppression presents a significant challenge in managing sepsis, as patients may simultaneously display ongoing inflammatory responses and compromised immune function [5]. Septic shock is a severe progression of sepsis marked by profound circulatory, cellular, and metabolic abnormalities. One of the defining characteristics of septic shock is significant hemodynamic instability. Patients experience widespread vasodilation, which decreases systemic vascular resistance and contributes to hypotension. In the early stages, the body may compensate with an increased cardiac output to counterbalance the decreased vascular resistance; however, this compensatory mechanism often fails as myocardial function deteriorates over time [6]. Even with potentially increased cardiac output, microcirculatory dysfunction often leads to inadequate tissue perfusion and oxygen delivery, exacerbating organ dysfunction. These hemodynamic alterations are pivotal in the progression of septic shock and underscore the importance of rapid intervention to restore adequate tissue perfusion and oxygenation to vital organs [6]. Oxygen is vital in cellular metabolism and tissue perfusion, which becomes critically important during sepsis. Under normal conditions, oxygen is delivered to tissues to support aerobic metabolism, producing adenosine triphosphate (ATP) necessary for cellular function. However, in sepsis, the balance between oxygen supply and demand is frequently disrupted [7]. Despite normal arterial oxygen levels, patients may suffer from tissue hypoxia due to impaired microcirculation and a reduced capacity for cellular oxygen utilization. This oxygen supply-demand mismatch forces a shift toward anaerobic metabolism, leading to lactic acidosis and further cellular injury. Therefore, ensuring adequate oxygen delivery and utilization is crucial for improving cellular function and aiding recovery in sepsis patients [7]. The oxygen supply-demand imbalance is a hallmark of sepsis and septic shock, driven by increased metabolic demand and decreased oxygen delivery. Sepsis often elevates metabolic demand due to an intensified immune response, increasing tissue oxygen consumption [8]. At the same time, hemodynamic instability, vasodilation, and microcirculatory dysfunction reduce effective oxygen delivery to tissues. Additionally, mitochondrial dysfunction in sepsis impairs cells' ability to utilize oxygen efficiently, even when oxygen is available. This imbalance significantly contributes to organ dysfunction and represents a critical target for therapeutic intervention in sepsis and septic shock. Approaches to optimizing oxygen delivery and enhancing cellular metabolism are key components in effectively managing sepsis [9].

Hyperoxia: definition and mechanisms

Hyperoxia is characterized by an abnormally high oxygen level in the tissues and organs, resulting from an excess oxygen supply beyond normal physiological requirements [10]. This condition is typically identified when the partial pressure of oxygen (PaO_2_) exceeds 100 mmHg, although some studies consider hyperoxia at levels above 120 mmHg or even 150 mmHg. Hyperoxia can occur in settings such as normobaric environments, where patients receive supplemental oxygen at normal atmospheric pressure, and hyperbaric conditions, where oxygen is administered at increased atmospheric pressure. While oxygen therapy is essential for treating hypoxia, the therapeutic window for oxygen administration is narrow. Hyperoxia, particularly in critically ill patients, can lead to adverse effects if not carefully managed [11]. One of the primary mechanisms of hyperoxia-related cellular and tissue injury is the increased production of ROS. Under normal conditions, ROS are vital for cell signaling, immune responses, and maintaining homeostasis. However, excessive oxygen levels can dramatically increase ROS production, leading to oxidative stress [11]. Oxidative stress damages crucial cellular components, including lipids, proteins, and DNA. For example, lipid peroxidation can compromise cell membrane integrity, and oxidative damage to proteins can disrupt their normal functions. In severe cases, this oxidative damage can initiate a cascade of inflammatory responses, contributing to tissue injury and worsening conditions such as acute respiratory distress syndrome (ARDS) and MODS [12].

**Figure 1:**
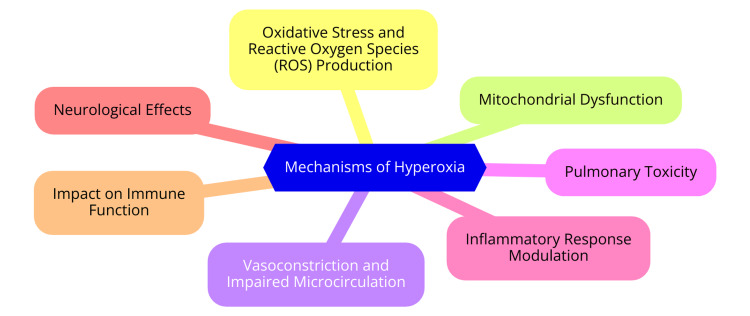
Mechanisms of hyperoxia Image Credit: Sharayu Paunikar

Hyperoxia also affects mitochondrial function and cellular respiration significantly. Mitochondria, the energy-producing organelles in cells, depend on a precise oxygen balance to generate ATP through oxidative phosphorylation. Excessive oxygen levels can cause mitochondrial dysfunction, reducing ATP production and increasing ROS generation [12]. This mitochondrial dysfunction can impair cellular energy metabolism, especially in tissues with high metabolic demands, like the heart and brain. The resulting energy deficit can cause cell injury and apoptosis, further contributing to organ dysfunction. Additionally, impaired mitochondrial function can exacerbate the inflammatory response, creating a vicious cycle of injury and inflammation [13]. Another critical aspect of hyperoxia is its vascular effects, particularly vasoconstriction and impaired microcirculation. Elevated oxygen levels can induce vasoconstriction in various vascular beds, including the cerebral and coronary circulations, reducing blood flow and compromising oxygen delivery to tissues despite high blood oxygen levels [14]. In critically ill patients, this phenomenon can exacerbate tissue hypoxia and contribute to organ dysfunction. Moreover, impaired microcirculation due to hyperoxia can hinder the exchange of nutrients and waste products at the cellular level, further complicating the clinical picture and potentially leading to multi-organ failure [15]. Despite the potential risks associated with hyperoxia, there are protective effects that warrant consideration. One of the primary benefits of elevated oxygen levels is their ability to enhance antimicrobial activity. High oxygen concentrations can improve immune cells' bactericidal capacity, such as neutrophils, by increasing ROS production, which is crucial for killing pathogens. This effect is particularly relevant in sepsis, where hyperoxia may bolster the immune response against infections and improve clinical outcomes [16]. Additionally, hyperoxia has been shown to modulate immune responses, potentially reducing inflammation in certain contexts. For example, hyperoxic environments may help downregulate pro-inflammatory cytokines in some inflammatory conditions and promote a more balanced immune response. However, the balance between these protective effects and the potential for oxidative injury remains complex and context-dependent, emphasizing the need for careful consideration when administering oxygen therapy in clinical practice [16]. Figure 1 illustrates the mechanisms of hyperoxia.

Clinical evidence of hyperoxia in sepsis and septic shock

Observational studies and retrospective analyses have significantly contributed to our understanding of hyperoxia's effects on patients with sepsis and septic shock. These studies, which often involve large patient cohorts, reveal the complex relationship between hyperoxia and patient outcomes. For instance, a systematic review of 12 studies with 15,782 patients highlighted this complexity. Six studies indicated an increased risk of mortality associated with hyperoxia, suggesting that excessive oxygen could be harmful in critically ill patients [17]. Conversely, three studies found no significant difference in mortality between hyperoxic therapy and normoxic levels, while one study even suggested a potential protective effect of hyperoxia. These discrepancies underline the variability in patient responses and the challenges in defining optimal oxygenation strategies for septic patients, given the differing definitions and thresholds for hyperoxia across studies [17]. Randomized controlled trials (RCTs) offer a more controlled approach to evaluating the impact of hyperoxia in sepsis and septic shock. For example, a post-hoc analysis of the HYPERS2S study found that hyperoxemia was associated with increased 90-day mortality in sepsis patients, raising concerns about high oxygen concentrations [18]. Conversely, another post-hoc analysis from the intensive care unit randomized oxygenation (ICU-ROX) study suggested that conservative oxygen therapy might be linked to higher mortality rates, complicating the clinical landscape. These conflicting results highlight the ongoing debate over optimal oxygen concentrations for sepsis and septic shock patients. Variations in trial designs, patient populations, and definitions of hyperoxia contribute to the complexity of drawing definitive conclusions [18]. Meta-analyses and systematic reviews provide a broader perspective by synthesizing evidence from multiple studies. A recent meta-analysis of 17 RCTs involving over 8,000 patients found that perioperative hyperoxia (a fraction of inspired oxygen, or FiO_2_, of 0.80) was associated with reduced risk of surgical site infections and overall mortality in major surgery [19]. However, this evidence does not specifically focus on sepsis, and the findings do not offer clear guidance on optimal oxygen management for septic patients. While hyperoxia may offer benefits in certain contexts, its use in septic patients remains contentious. The potential for oxidative stress and organ dysfunction associated with high oxygen levels necessitates a careful, individualized approach to oxygen therapy [19].

Therapeutic implications of hyperoxia in sepsis management

The management of sepsis and septic shock requires a nuanced approach to oxygen therapy, particularly when considering the use of hyperoxia. Research indicates mixed outcomes regarding hyperoxia in septic patients. A systematic review of 10 studies revealed varied results: six reported higher mortality rates associated with hyperoxia, three found no significant difference, and one suggested a reduced risk of mortality with hyperoxia [20]. The HYPERS2S trial further emphasized concerns, indicating that elevated arterial oxygen levels might increase mortality risk in septic shock patients, especially when these levels exceed normal thresholds. Additionally, hyperoxia has been associated with systemic impairments, potentially exacerbating tissue damage and organ dysfunction and leading to complications such as acute lung injury (ALI) and prolonged ICU stays [20]. The debate over optimal oxygen targets in sepsis management continues, with conflicting evidence on the benefits of hyperoxia versus conservative oxygen therapy. Hyperoxia can enhance bactericidal activity and potentially stabilize hemodynamics, posing increased mortality risks and organ dysfunction. Excessive oxygen levels can be particularly harmful in vulnerable populations, such as the elderly or those with chronic respiratory conditions [17]. In contrast, conservative oxygen therapy may mitigate some risks but may also be associated with its challenges, including potential adverse effects. Current guidelines from the Surviving Sepsis Campaign recommend cautious use of oxygen, emphasizing maintaining oxygen levels within a safe range (PaO_2_ between 60 and 240 mmHg) to avoid hypoxia and hyperoxia [21]. The campaign acknowledges insufficient evidence to endorse specific conservative targets for oxygen saturation or partial pressure of oxygen. Various critical care societies align with this stance, advocating for individualized oxygen therapy based on patient-specific factors and avoiding hyperoxia in high-risk patients, particularly those with pre-existing pulmonary conditions. In summary, while hyperoxia might offer certain benefits, its potential for adverse effects necessitates careful monitoring and adjustment of oxygen therapy in sepsis and septic shock management. The consensus is to avoid excessive oxygen levels and tailor oxygen therapy to individual patient needs, balancing the benefits and risks to optimize outcomes [21]. The therapeutic implications of hyperoxia in sepsis management are summarized in Table 1.

**Table 1:** Therapeutic implications of hyperoxia in the management of sepsis and septic shock

Aspect	Implication	Details
Oxygen target levels [22]	Establishing optimal oxygen saturation targets to avoid hyperoxia	Maintaining oxygen saturation (SpO_2_) levels between 92% and 96% to balance adequate oxygenation with minimizing the risk of hyperoxia
Clinical outcomes [23]	Impact on mortality, organ dysfunction, and length of ICU stay	Hyperoxia is associated with increased mortality and risk of organ damage due to oxidative stress and impaired microcirculation
Guideline recommendations [24]	Adopting conservative oxygen therapy strategies	Guidelines suggest titrating oxygen to avoid excessive levels, particularly in patients without severe hypoxemia
Pulmonary management [24]	Mitigating hyperoxia-induced lung injury	Careful oxygen titration is needed to prevent pulmonary toxicity and acute lung injury from prolonged exposure to high oxygen levels
Individualized therapy [25]	Personalizing oxygen therapy based on patient-specific factors	Adjusting oxygen therapy considering factors like baseline oxygenation status, comorbidities, and response to treatment
Continuous monitoring [26]	Implementing close monitoring of oxygen levels and clinical parameters	Using pulse oximetry and arterial blood gas analysis to monitor oxygen levels and adjust therapy dynamically
Research and development [27]	Encouraging further research on optimal oxygenation strategies and biomarkers to guide therapy	Identifying biomarkers for oxidative stress and developing protocols for individualized oxygen therapy in sepsis management

Risks and adverse effects of hyperoxia

Hyperoxia, characterized by an excessive amount of oxygen in the tissues, poses several potential risks that can adversely affect various organ systems. One of the most concerning effects is pulmonary toxicity. High oxygen levels can lead to oxidative damage in lung tissues [10]. This damage may manifest as ALI or ARDS, conditions resulting from inflammation and cell injury triggered by increased formation of ROS. The resulting inflammation can compromise the integrity of the alveolar-capillary membrane, leading to pulmonary edema and impaired gas exchange, complicating the management of critically ill patients [28]. In addition to pulmonary effects, hyperoxia can have significant cardiovascular consequences. Elevated oxygen levels can alter myocardial function by reducing cardiac output and stroke volume while increasing systemic vascular resistance. These physiological changes can lead to decreased coronary blood flow and myocardial oxygen consumption, raising the risk of reperfusion injury due to heightened ROS production. Observational studies have linked hyperoxia to increased myocardial injury, particularly in patients with conditions such as myocardial infarction, and have suggested a correlation with higher mortality rates among critically ill patients [28]. The neurological risks associated with hyperoxia are also significant. Elevated oxygen levels can cause hyperoxic brain injury, characterized by cerebral vasoconstriction and mitochondrial dysfunction, impairing oxygen delivery to brain tissues [29]. This impairment may result in neuronal damage and cognitive dysfunction, particularly concerning patients with pre-existing conditions such as traumatic brain injury. Prolonged exposure to hyperoxia has been associated with cognitive deficits and an increased risk of neurodegenerative processes, highlighting the potential long-term impacts of excessive oxygen exposure on brain health [30]. The risks and adverse effects of hyperoxia in sepsis and septic shock are summarized in Table 2.

**Table 2:** Risks and adverse effects of hyperoxia in sepsis and septic shock

Category	Risks and adverse effects	Mechanisms	Clinical manifestations
Pulmonary [31]	Hyperoxia-induced lung injury	Oxidative stress leads to alveolar epithelial cell damage, inflammation, and impaired surfactant production	Acute lung injury, atelectasis, reduced lung compliance, and worsening of acute respiratory distress syndrome (ARDS)
Cardiovascular [31]	Impaired myocardial function, increased vascular resistance, decreased cardiac output	Oxygen-induced vasoconstriction reduced coronary blood flow and impaired endothelial function	Myocardial ischemia, arrhythmias, increased blood pressure, and reduced cardiac efficiency
Neurological [32]	Hyperoxic brain injury, cognitive dysfunction	Reduced cerebral blood flow due to oxygen-induced vasoconstriction and oxidative damage to neural tissue	Confusion, delirium, cognitive decline, and potential exacerbation of neurological conditions
Renal [32]	Kidney injury and dysfunction	Oxidative damage to renal tubular cells, microvascular dysfunction, and reduced renal perfusion	Acute kidney injury, reduced glomerular filtration rate, and electrolyte imbalances
Hematological [33]	Impaired red blood cell deformability, increased risk of thrombosis	Oxidative stress causes red blood cell damage and endothelial injury, leading to a pro-thrombotic state	Increased risk of thromboembolic events and impaired oxygen delivery due to altered red blood cell function
Gastrointestinal [33]	Mucosal injury and impaired gut barrier function	Reduced splanchnic blood flow and oxidative stress damage the gut mucosa	Gastrointestinal bleeding, increased permeability leading to bacterial translocation and infection risk

Special populations and considerations

Several limitations characterize the existing literature on hyperoxia in sepsis and septic shock. Many studies are retrospective or observational, restricting the ability to draw definitive conclusions about causality. The number of RCTs is limited, and those available often have methodological issues, such as small sample sizes and inadequate adjustment for confounding variables [17]. Additionally, there is substantial variability in patient populations across studies, including differences in underlying conditions, infection types, and treatment protocols. This heterogeneity complicates result interpretation and the generalizability of findings to different clinical settings. Furthermore, the lack of standardization in defining hyperoxia impedes effective comparison of results across studies. Variations in thresholds for arterial oxygen levels (PaO_2_) and the duration of hyperoxia exposure exacerbate this issue [34]. To address these gaps in understanding hyperoxia's role in sepsis management, several areas warrant further investigation. Future research should aim to elucidate the underlying mechanisms by which hyperoxia influences sepsis outcomes. Examining how different oxygen levels affect immune responses, bacterial sensitivity to antibiotics, and cellular metabolism could provide insights into optimizing oxygen therapy. Identifying the most effective oxygenation strategies is crucial, including determining the optimal range of oxygenation that balances risks and therapeutic benefits. This research should consider how oxygen levels might need adjustment based on specific pathogens and the severity of the infection [20]. The complexity of sepsis calls for a shift toward personalized medicine, particularly in oxygen therapy. Tailoring oxygen administration to individual patient characteristics, including their physiological response to sepsis, could enhance outcomes. Personalized approaches might involve using biomarkers to guide therapy, ensuring patients receive the most appropriate oxygen levels for their specific condition. Biomarkers could be instrumental in optimizing oxygen therapy for sepsis [35]. Identifying specific biomarkers related to sepsis severity, tissue oxygenation status, and inflammatory response may assist clinicians in making informed decisions about oxygen administration. This could lead to more effective management strategies considering each patient's unique biological responses, potentially reducing the risks associated with both hypoxia and hyperoxia [36].

Future directions and research gaps

The existing literature on hyperoxia in sepsis and septic shock reveals several limitations. Many studies are retrospective or observational, which constrains the ability to establish causality definitively. RCTs are scarce, and those that exist often suffer from methodological flaws, such as small sample sizes and insufficient adjustment for confounding variables. Additionally, these studies frequently show considerable variability in patient populations, including differences in underlying conditions, types of infections, and treatment protocols [17]. This heterogeneity complicates the interpretation of results and the generalizability of findings across various clinical settings. Furthermore, the lack of standardization in defining hyperoxia impedes effective comparison across studies. Variations in thresholds for arterial oxygen levels (PaO_2_) and the duration of hyperoxia exposure exacerbate this issue [37]. To address these gaps in understanding hyperoxia's role in sepsis management, several areas require further investigation. Future research should explore the mechanisms by which hyperoxia affects sepsis outcomes. Examining how different oxygen levels impact immune responses, bacterial sensitivity to antibiotics, and cellular metabolism could provide valuable insights into optimizing oxygen therapy. Identifying the most effective oxygenation strategies is crucial, including determining the optimal range of oxygenation that balances risks and therapeutic benefits. This involves understanding how oxygen levels might need adjustment based on specific pathogens and the severity of the infection [38]. The complexity of sepsis highlights the need for a shift toward personalized medicine, particularly in oxygen therapy. Tailoring oxygen administration based on individual patient characteristics, including their physiological response to sepsis, could enhance outcomes. Personalized approaches might involve using biomarkers to guide therapy, ensuring patients receive the most appropriate oxygen levels for their specific condition. Biomarkers could be instrumental in optimizing oxygen therapy for sepsis [39]. Identifying biomarkers related to sepsis severity, tissue oxygenation status, and inflammatory response may aid clinicians in making informed decisions about oxygen administration. This approach could lead to more effective management strategies considering each patient's unique biological responses, potentially reducing the risks associated with both hypoxia and hyperoxia [40]. Future directions and research gaps in hyperoxia management for sepsis and septic shock are summarized in Table 3.

**Table 3:** Future directions and research gaps in hyperoxia management for sepsis and septic shock

Research area	Description	Objectives
Optimal oxygenation targets [41]	Determining the safest and most effective oxygen saturation levels for sepsis and septic shock patients	To identify specific oxygenation thresholds that balance sufficient tissue oxygenation to minimize hyperoxia-related harm
Mechanistic studies on hyperoxia [41]	Investigating the cellular and molecular mechanisms of hyperoxia-induced injury in septic patients	To understand how hyperoxia contributes to oxidative stress, mitochondrial dysfunction, and microcirculatory impairment
Personalized oxygen therapy [42]	Developing individualized oxygen therapy protocols based on patient characteristics such as comorbidities and disease severity	To optimize oxygen delivery by tailoring therapy to the unique physiological needs of each patient, potentially improving outcomes
Pediatric and geriatric populations [43]	Assessing the effects of hyperoxia and optimal oxygen strategies in special populations like children and the elderly	To establish evidence-based guidelines that address these groups' specific needs and vulnerabilities in sepsis management
Long-term outcomes of oxygen therapy [43]	Evaluating the impact of different oxygenation strategies on long-term outcomes, including neurocognitive function and quality of life	To provide a more comprehensive understanding of the benefits and risks associated with oxygen therapy beyond immediate survival
Biomarkers for oxygen therapy guidance [44]	Identifying biomarkers to predict patient response to oxygen therapy and guide its administration	To enable more precise and responsive adjustments to oxygen therapy, improving patient safety and efficacy
Comparative studies of oxygen strategies [45]	Comparing conservative oxygen therapy to liberal oxygenation strategies in large, multicenter randomized controlled trials	To provide robust data on the clinical effectiveness and safety of different oxygenation strategies in diverse ICU settings
Cost-effectiveness analyses [46]	Analyzing the cost-effectiveness of various oxygen therapy approaches in sepsis management	To guide healthcare resource allocation and improve the value of care delivered to septic patients

## Conclusions

In conclusion, while oxygen therapy remains a cornerstone in the management of sepsis and septic shock, the role of hyperoxia warrants careful consideration due to its complex and potentially harmful effects. The clinical evidence suggests that while maintaining adequate oxygenation is essential to prevent tissue hypoxia, excessive oxygen levels can lead to oxidative stress, impair microcirculation, and negatively affect outcomes in septic patients. The balance between providing sufficient oxygen to meet metabolic demands and avoiding the detrimental effects of hyperoxia is delicate, highlighting the need for individualized oxygen therapy strategies. Current guidelines and studies advocate for cautious titration of oxygen to avoid both hypoxia and hyperoxia. Still, more research is needed to determine the optimal oxygenation targets for different patient populations. Future studies should focus on refining oxygen therapy protocols, identifying specific patient subgroups that may benefit from tailored oxygenation strategies, and exploring biomarkers to guide therapy. Ultimately, a nuanced approach to oxygen management in sepsis and septic shock will likely improve patient outcomes and reduce the incidence of oxygen-related complications.
